# Supplementary material for: Total genetic contribution assessment across the human genome
Source: Nat Commun. 2021 May 14;12:2845. doi: 10.1038/s41467-021-23124-w (PMC8121943; doi:10.1038/s41467-021-23124-w)
Supplement: Supplementary file 1 — Supplementary Information [file 41467_2021_23124_MOESM1_ESM.pdf]

*Supplementary Information*

*for*

# Total genetic contribution assessment across the human genome

by Ting Li, Zheng Ning, Zhijian Yang, Ranran Zhai, Chenqing Zheng, Wenzheng Xu, Yipeng Wang, Kejun Ying, Yiwen Chen, Xia Shen

## Supplementary Methods

**TGCA likelihood inference** For a single SNP, denote $\mathbf{z} = (z_1, \ldots, z_k)$ be an i.i.d. sample of GWAS $Z$ statistics of $k$ phenotypes, adjusted for phenotypic correlations, from the mixture model

$$\pi_- N(\mu_-, \sigma_1^2) + \pi_0 N(0,1) + \pi_+ N(\mu_+, \sigma_2^2),$$

with density

$$f_\theta(u) = \pi_- \phi_-(u|\mu_-, \sigma_1^2) + \pi_0 \phi(u) + \pi_+ \phi_+(u|\mu_+, \sigma_1^2)$$

where the three components correspond to the negative effects, the null effects, and the positive effects. $\phi(.)$ denotes the standard normal density and $\phi(.|\mu, \sigma^2)$ the normal density with mean $\mu$ and variance $\sigma^2$. The proportions of the components are constrained by $\pi_- + \pi_0 + \pi_+ = 1$. $\theta = (\pi_-, \pi_0, \pi_+, \mu_-, \mu_+, \sigma_1^2, \sigma_2^2)$ are the unknown parameters.

The log-likelihood based on the observed data $\mathbf{z}$ is

$$\log L(\theta; \mathbf{z}) = \sum_i^k \log f_\theta(z_i).$$

Due to the constraint $\pi_- + \pi_0 + \pi_+ = 1$, ordinary optimization algorithms such as the Newton–Raphson is prone to failure. An expectation-maximization (EM) algorithm is normally required.

Rewriting the log-likelihood, incorporating the latent variable $\boldsymbol{\beta}$, i.e., the vector of true genetic effects on each phenotype, we have

$$\begin{aligned}\log f(\mathbf{z}, \boldsymbol{\beta}|\theta) &= \sum_{i=1}^k \{ I(\beta_i < 0)(\log \pi_- + \log \phi(z_i|\mu_-, \sigma_1^2)) \\ &+ I(\beta_i = 0)(\log \pi_0 + \log \phi(z_i)) \\ &+ I(\beta_i > 0)(\log \pi_+ + \log \phi(z_i|\mu_+, \sigma_2^2))\}\end{aligned}$$

The expected full log-likelihood is:

$$\begin{aligned}E_{\boldsymbol{\beta}|\mathbf{z}}[\log f(\mathbf{z}, \boldsymbol{\beta}|\theta)] &= \sum_{i=1}^k \{ E_{\boldsymbol{\beta}|\mathbf{z}}[I(\beta_i < 0)](\log \pi_- + \log \phi(z_i|\mu_-, \sigma_1^2)) \\ &+ E_{\boldsymbol{\beta}|\mathbf{z}}[I(\beta_i = 0)](\log \pi_0 + \log \phi(z_i)) \\ &+ E_{\boldsymbol{\beta}|\mathbf{z}}[I(\beta_i > 0)](\log \pi_+ + \log \phi(z_i|\mu_+, \sigma_2^2))\} \\ &= \sum_{i=1}^k \{ Pr(\beta_i < 0|\mathbf{z})(\log \pi_- + \log \phi(z_i|\mu_-, \sigma_1^2)) \\ &+ Pr(\beta_i = 0|\mathbf{z})(\log \pi_0 + \log \phi(z_i)) \\ &+ Pr(\beta_i > 0|\mathbf{z})(\log \pi_+ + \log \phi(z_i|\mu_+, \sigma_2^2))\}\end{aligned}$$

With these, an EM algorithm can proceed as:

**E-step:** Initiate $\theta$ and evaluate $Pr(\beta_i < 0|\mathbf{z})$, $Pr(\beta_i = 0|\mathbf{z})$, $Pr(\beta_i > 0|\mathbf{z})$.

**M-step:** Maximize the expected full log-likelihood above with respect to $\theta$.

The standard errors of the estimated $\theta$ were derived from the Hessian matrix using numerical procedures. Thereafter, assuming independence of $\hat{\pi}_-\hat{\mu}_-$ and $\hat{\pi}_+\hat{\mu}_+$, the variance of $\hat{\Theta}$ can be derived via the Delta method:

$$\begin{aligned}\mathrm{var}(\hat{\Theta}) &= \mathrm{var}(\hat{\pi}_-\hat{\mu}_-) + \mathrm{var}(\hat{\pi}_+\hat{\mu}_+) \\ &\approx \hat{\pi}_-^2\mathrm{var}(\hat{\mu}_-) + \hat{\mu}_-^2\mathrm{var}(\hat{\pi}_-) + \mathrm{var}(\hat{\mu}_-)\mathrm{var}(\hat{\pi}_-) \\ &+ \hat{\pi}_+^2\mathrm{var}(\hat{\mu}_+) + \hat{\mu}_+^2\mathrm{var}(\hat{\pi}_+) + \mathrm{var}(\hat{\mu}_+)\mathrm{var}(\hat{\pi}_+)\end{aligned}$$

from which the standard error can be derived. See also implementation at `https://github.com/xiashen/TGCA`.

**Locus definition** The FUMA/SNP2GENE procedure [1] was used to define genomic loci based on genome-wide $\hat{\Theta}$. We report those top loci that had $\hat{\Theta} > 2$ and independent of each other with $r^2 < 0.1$. Independent significant SNPs that are in LD with each other ($r^2 \geqslant 0.1$) were assigned to the same genomic locus. Independent significant SNPs that are closer than 250 kb were merged into one locus.

**Gene-based total genetic contribution statistics** We obtained the gene-based p-values of TGCA by the FUMA/SNP2GENE procedure (**Supplementary Data 13-17**), which uses the input p-values for $\hat{\Theta}$ to compute gene-based p-values using the MAGMA tool [2]. The gene-based p-values were computed for protein-coding genes by mapping SNPs to genes if SNPs are located within the annotated gene windows (1 kb window on both sides). For the analyses, the default MAGMA setting (SNP-wise model for gene analysis) were used, and the Bonferroni correction (gene) was used to correct for multiple testing. 1000 Genomes phase 3 data were used as a reference panel to assess LD across SNPs and genes.

**Gene set enrichment of total genetic contribution** SNPs in LD ($r^2 > 0.6$) of top SNPs were annotated to genes through ANNOVAR [3], and we only chose protein-coding genes for mapping. FUMA/GENE2FUNC [1] then used these prioritised genes as input to check expression patterns and shared molecular functions between genes(**Supplementary Data 18-20**). To test for overrepresentation of biological functions, the prioritised genes were tested against the gene sets obtained from MsigDB [4] and WikiPathways [5] using hypergeometric tests. Multiple testing correction is performed over the gene sets. FUMA reported the gene sets with adjusted p-values less than 0.05 and the number of genes that overlap with the gene set larger than 1.

**Simulations of TGCA with different LD levels** To assess the relationship between $\hat{\Theta}$ correlations and LD correlations, we considered 21 different level of LD with $r = 0, 0.05, 0.1, 0.15, \cdots, 1$. For each pre-defined LD correlation, we repeated the simulation for 10 times. In each simulation, genotypes for two SNPs and 100,000 individuals were generated assuming the given LD correlation, and 200 independent true genetic effects corresponding to 200 phenotypes were drawn from $N(0, 1)$ and assigned to one of the two SNPs. We conducted an association analysis to obtain 200 Z-scores for the 200 traits for both SNPs and ran TGCA to estimate $\Theta$. This procedure were repeated for 200 times to generate 200 pairs of $\hat{\Theta}$ to calculate the $\hat{\Theta}$ correlation between the two SNPs.

**Functional enrichment analysis of TGCA** Similar to the strategy used by the HOPS method [6], we used a SNP-based regression with LD-corrected $\hat{\Theta}$ to estimate the effect of each genomic functional annotation on $\hat{\Theta}$. We considered the 28 inbuilt primary functional annotations in `ldsc` [7]. For each tissue and each phenotype domain:

$$\hat{\Theta}_j = \alpha + \delta \ell_j + \gamma F_j + \epsilon_j$$

where $\ell_j$ is the LD score of the $j$-th SNP, pre-calculated by the `ldsc` software; $F_j$ takes a value of zero or one, as an indicator for whether the SNP is annotated within a particular functional region; $\gamma$ is the parameter of interest. The more the functional annotation can explain the variation in $\hat{\Theta}$, the more positive $\gamma$ would be. As LD exists across the analysed SNPs, directly applying the regression to all the SNPs would underestimate var($\hat{\gamma}$). We split the SNPs into 100 subsets, where each subset contained SNPs $j, j + 100, j + 200, \cdots$, $j = 1, 2, \cdots, 100$, so that the LD correlations were pruned. These resulted in 100 $\hat{\gamma}$ estimates, and we report the median of them.

## Supplementary Results

**Connection between TGCA and linkage disequilibrium** We extracted the pre-computed LD scores using 1000 Genomes European ancestry data from the LD score regression software `ldsc` [8], where the SNPs all have MAF $> 0.05$, with the MHC region excluded. 399,817 SNPs that overlapped with our TGCA genome scans were passed onto subsequent investigation. For each phenotype domain, we conducted a Pearson's correlation test to examine the relationship between $\hat{\Theta}$ and the LD scores (**Supplementary Fig. 20**). Except for the diet phenotypes, $\hat{\Theta}$ had significant positive correlations with the LD scores in the other trait domains.

**Gene-gene interactions and pathways** We extracted 6,979,630 established gene-gene interactions in the GeneMANIA database [9,10] and 9,549 biological pathways containing different numbers of coding genes in the MSigDB database [4,11] for the TGCA assessed coding genes (**Supplementary Data 13-17**). For diet and mental health traits, we found that the number of genes that a gene inter-

acts with was positively correlated with the gene-based $\Theta$ statistic ($-\log_{10} P$) (**Supplementary Fig. 21a**), and for a given gene, the number of pathways that had at least one gene interacting with it also showed similar positive correlation (**Supplementary Fig. 21b**). Although the correlation estimates were low, they suggest that $\Theta$ has information about pleiotropy, for which the more pleiotropic a gene is, the more it tends to be involved in more interactions with other genes and pathways.

**Association between TGCA and genomic functional annotations** For the 28 functional annotations, we used the LD-correction regression models to estimate the effect of each annotation on the magnitude of $\hat{\Theta}$ for each trait domain (**Supplementary Fig. 22**). Similar to what we did for the association between $\hat{\Theta}$ and tissue-specifically expressed genes, we also tested for enrichment of $\hat{\Theta}$ on these functional annotations using stratified LD score regression (not shown). However, poor correlations were observed between the resulted scores from the two methods, indicating most of the results were too noisy to be trustworthy. Nevertheless, the results did highlight the transcription start sites (TSS) to be the strongest association with $\hat{\Theta}$ in the physical measures domain of phenotypes.

# Supplementary Figures

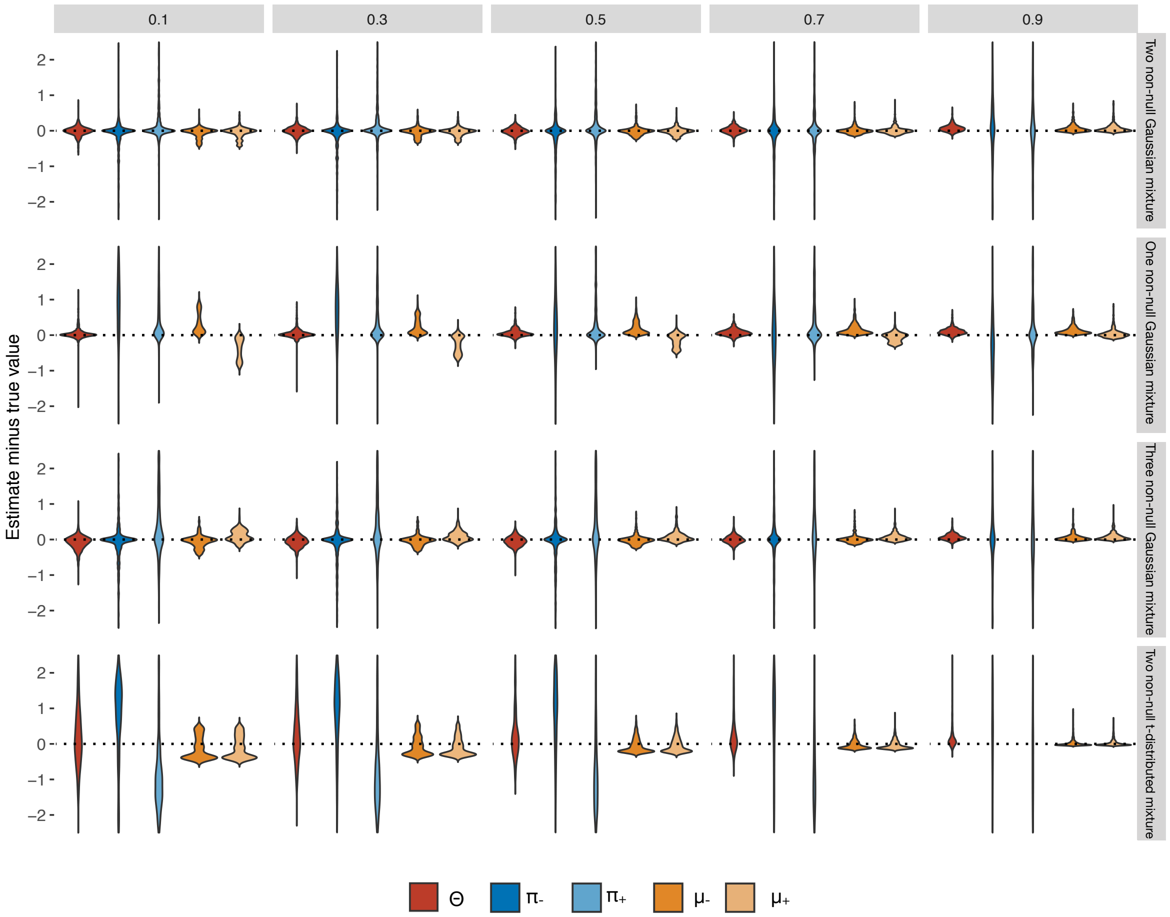


**Supplementary Figure 1: Simulation results under different true models.** 200 independent Z-scores for a single genetic variant were simulated from a mixture distribution with two non-null Gaussian components $\pi_- N(\mu_-, \sigma_1^2) + \pi_0 N(0,1) + \pi_+ N(\mu_+, \sigma_2^2)$, where the $\pi_- = \pi_+$; a mixture with one non-null Gaussian $\pi_0 N(0,1) + \pi_+ N(\mu_+, \sigma^2)$; a mixture with three non-null Gaussian $\pi_- N(\mu_-, \sigma_1^2) + \pi_0 N(0,1) + \pi_+ N(\mu_+, \sigma_2^2) + \pi_{++} N(\mu_{++}, \sigma_3^2)$, where $\pi_- = 2\pi_+ = 2\pi_{++}$; and a mixture with two non-null t-distributed heavy-tailed components $\pi_-[t(1) + \mu_-] + \pi_0 N(0,1) + \pi_+[t(1) + \mu_+]$, where $t(1)$ denotes a t-distribution with 1 degree of freedom, and $\pi_- = \pi_+$. Five different proportions of null effect $\pi_0$ were considered, ranging from 0.1 to 0.9. The simulation was repeated for 999 times. In each simulation, the negative effect size $\mu_-$ was randomly drawn from -$|N(1,1)|$, the positive effect size(s) $\mu_+$ and $\mu_{++}$ were drawn from $|N(1,1)|$, and the $\sigma^2$ parameters from $\chi^2(1)$. The y-axis compares the estimated parameters with the true values. Source data are provided as a Source Data file.

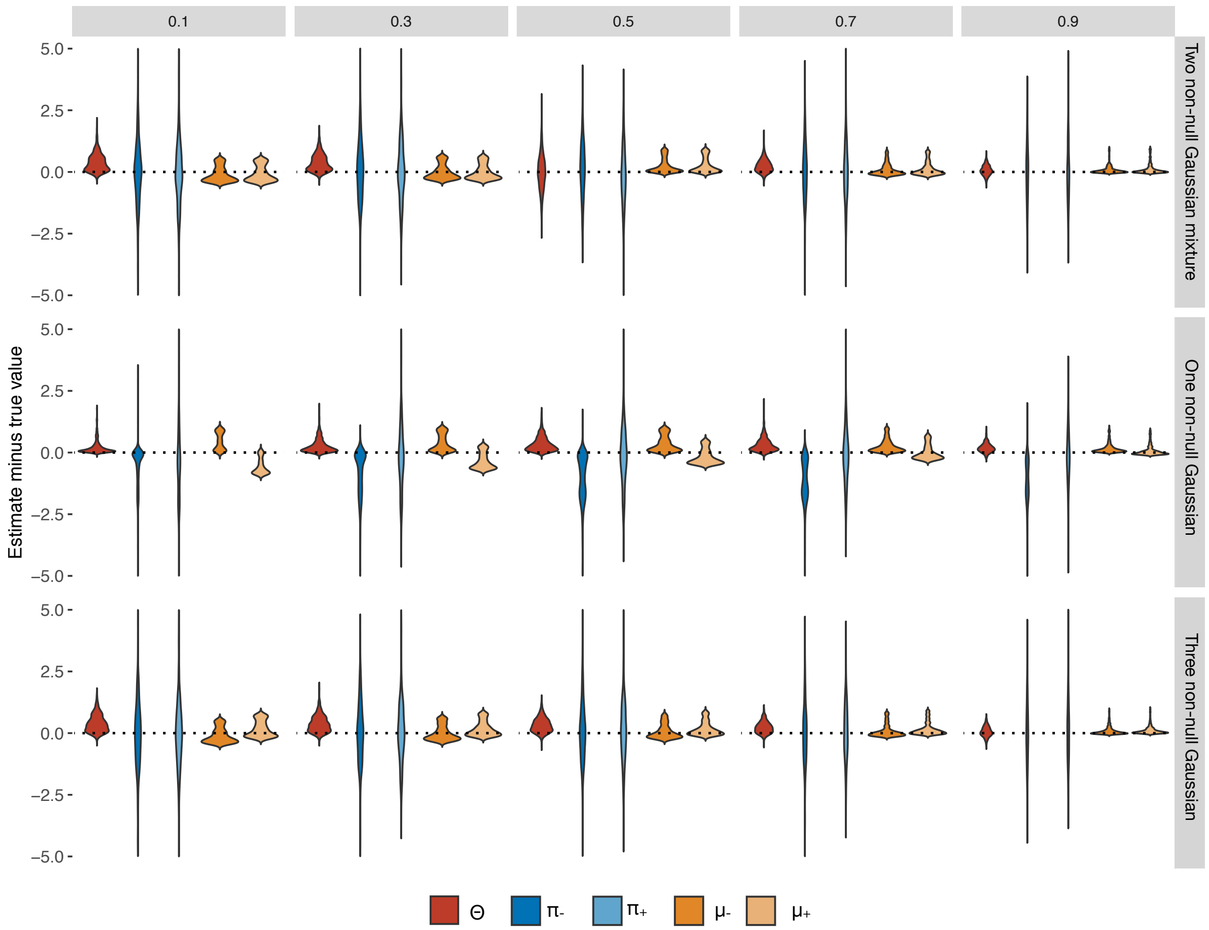


**Supplementary Figure 2: Simulation results under different true models using correlated Z-scores.** 1376 correlated Z-scores for a single genetic variant were simulated from a mixture distribution with two non-null Gaussian components $\pi_- N(\mu_-, \sigma_1^2) + \pi_0 N(0,1) + \pi_+ N(\mu_+, \sigma_2^2)$, where the $\pi_- = \pi_+$; a mixture with one non-null Gaussian $\pi_0 N(0,1) + \pi_+ N(\mu_+, \sigma^2)$; a mixture with three non-null Gaussian $\pi_- N(\mu_-, \sigma_1^2) + \pi_0 N(0,1) + \pi_+ N(\mu_+, \sigma_2^2) + \pi_{++} N(\mu_{++}, \sigma_3^2)$, where $\pi_- = 2\pi_+ = 2\pi_{++}$. Five different proportions of null effect $\pi_0$ were considered, ranging from 0.1 to 0.9. The simulation was repeated for 999 times. In each simulation, 1376 Z-scores were decorrelated and regularised before applying TGCA. The negative effect size $\mu_-$ was randomly drawn from -$|N(1,1)|$, the positive effect size(s) $\mu_+$ and $\mu_{++}$ were drawn from $|N(1,1)|$, and the $\sigma^2$ parameters from $\chi^2(1)$. The y-axis compares the estimated parameters with the true values. Source data are provided as a Source Data file.

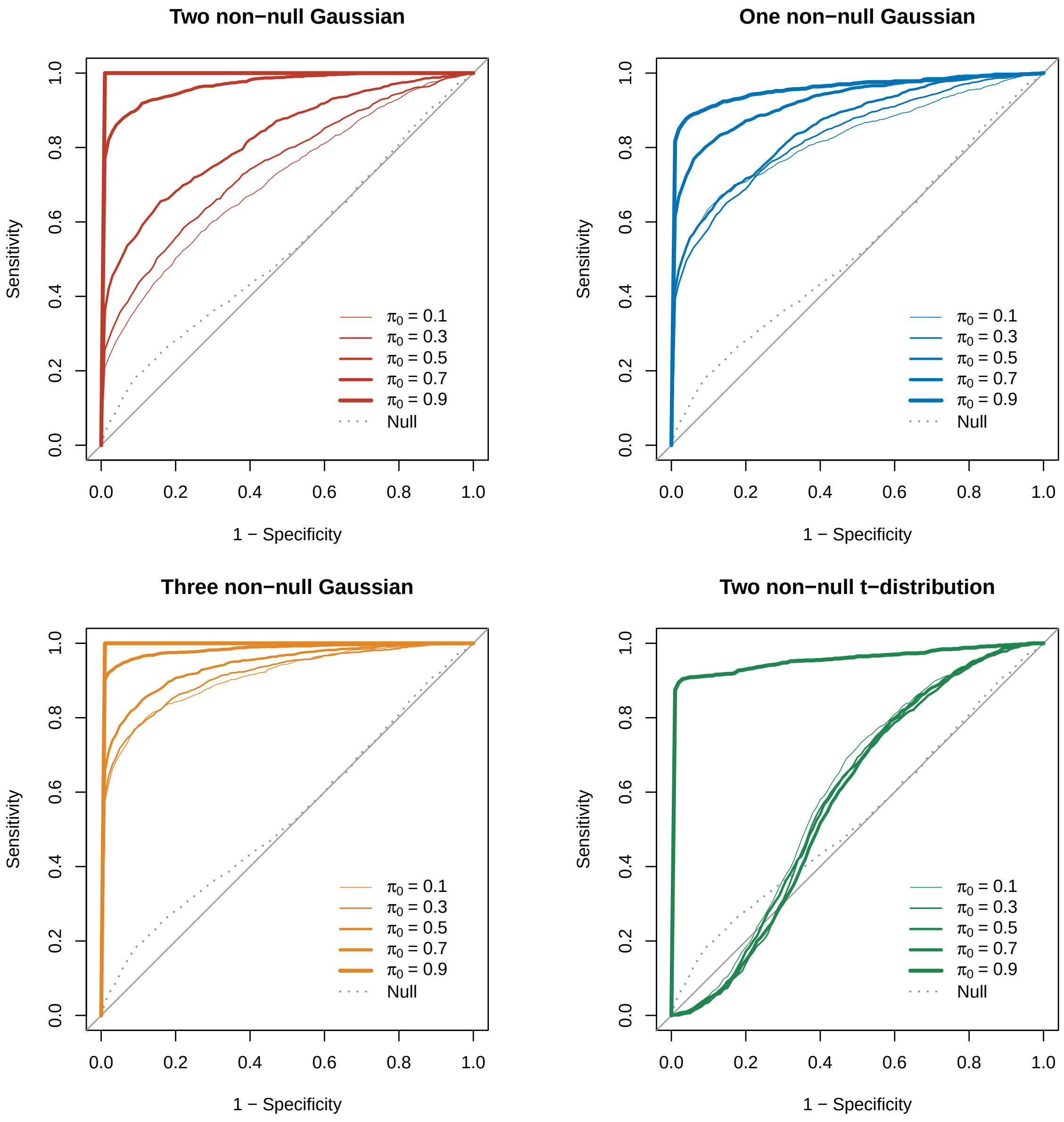


**Supplementary Figure 3: ROC curves of testing $\Theta = 0$ under different true models and parameter setups.** For each true model,$\Theta$ had a true value of 1, the null effect proportion $\pi_0$ ranged from 0.1 to 0.9. The grey point line shows the performance under the null. Each curve was produced via 999 repeats of the simulation. Source data are provided as a Source Data file.

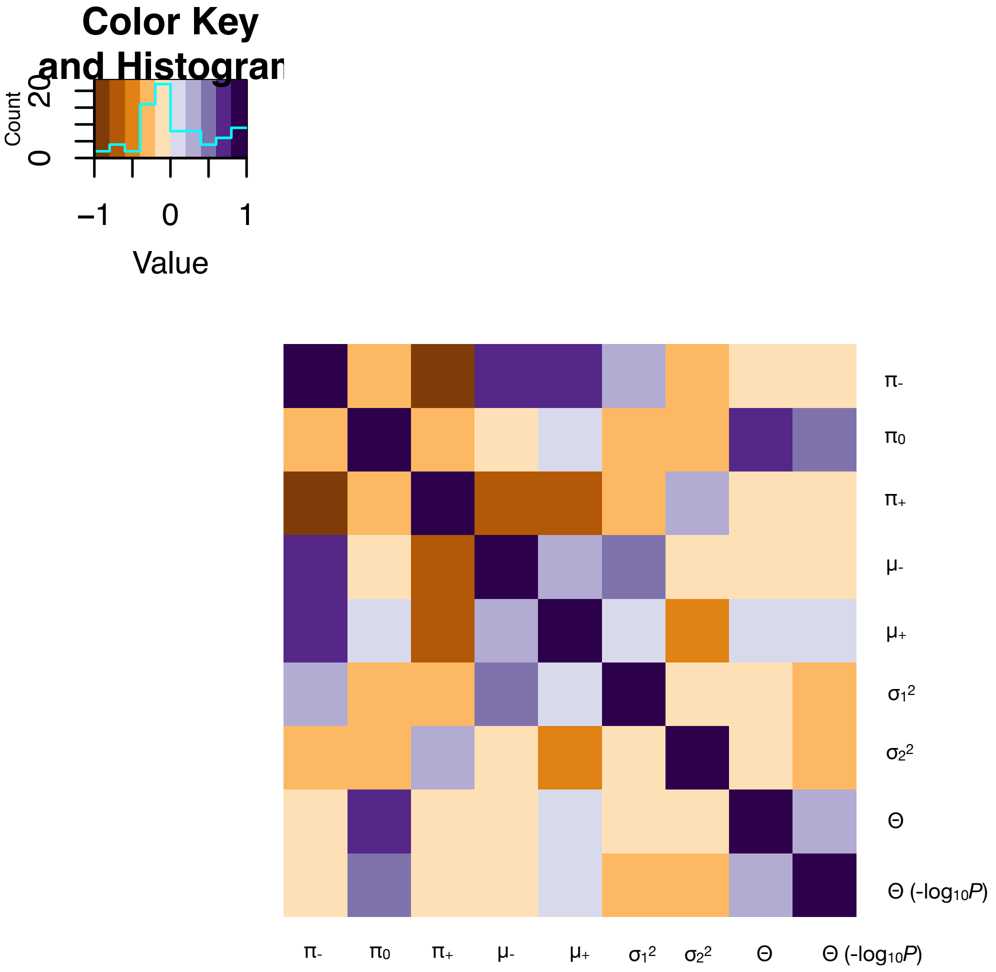


**Supplementary Figure 4: Correlation between estimated parameters in genome-wide TGCA in 1,376 UK Biobank phenotypes.** The labeled parameters correspond to $\pi_-$, $\pi_0$, $\pi_+$, $\mu_-$, $\mu_+$, $\sigma_1^2$, $\sigma_2^2$, $\Theta$, $-\log_{10} P_\Theta$, respectively. Correlation coefficients were estimated across genome-wide SNPs. Source data are provided as a Source Data file.

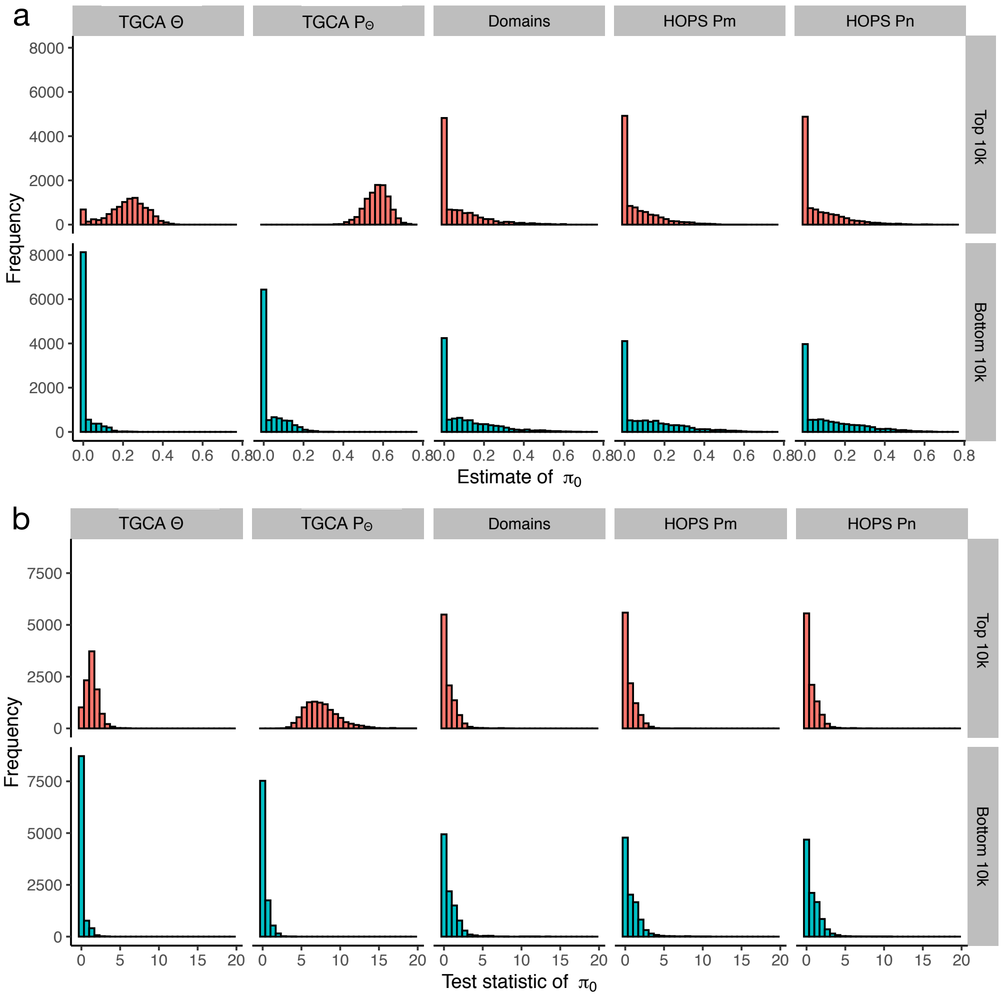


**Supplementary Figure 5: Comparison of $\hat{\pi}_0$ of top and bottom 10,000 SNPs ranked by five different statistics.** $P_\Theta$ values were derived from a two-sided Wald test against $\Theta = 0$. **a.** Distributions of $\pi_0$ estimates. **b.** Distributions of Wald test statistics against $\pi_0 = 0$. Source data are provided as a Source Data file.

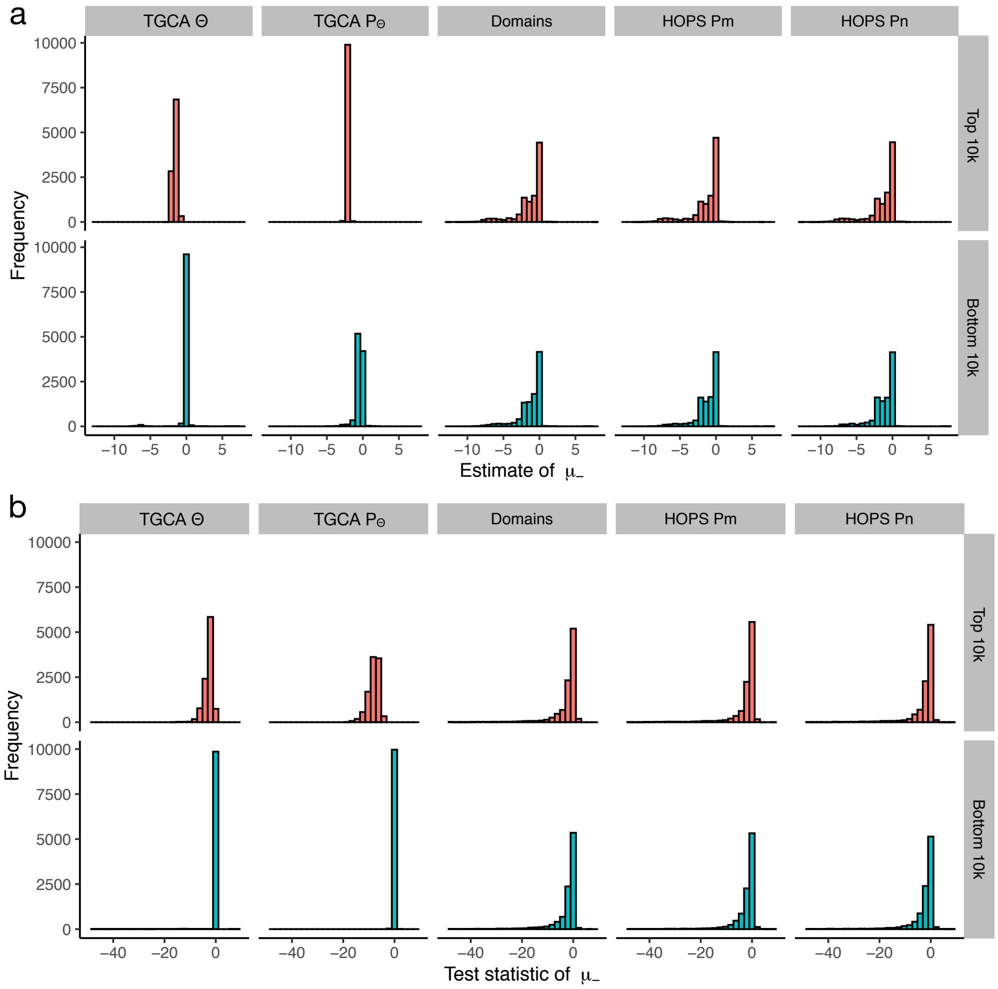


**Supplementary Figure 6: Comparison of $\hat{\mu}_-$ of top and bottom 10,000 SNPs ranked by five different statistics.** $P_\Theta$ values were derived from a two-sided Wald test against $\Theta = 0$. **a.** Distributions of $\mu_-$ estimates. **b.** Distributions of Wald test statistics against $\mu_- = 0$. Source data are provided as a Source Data file.

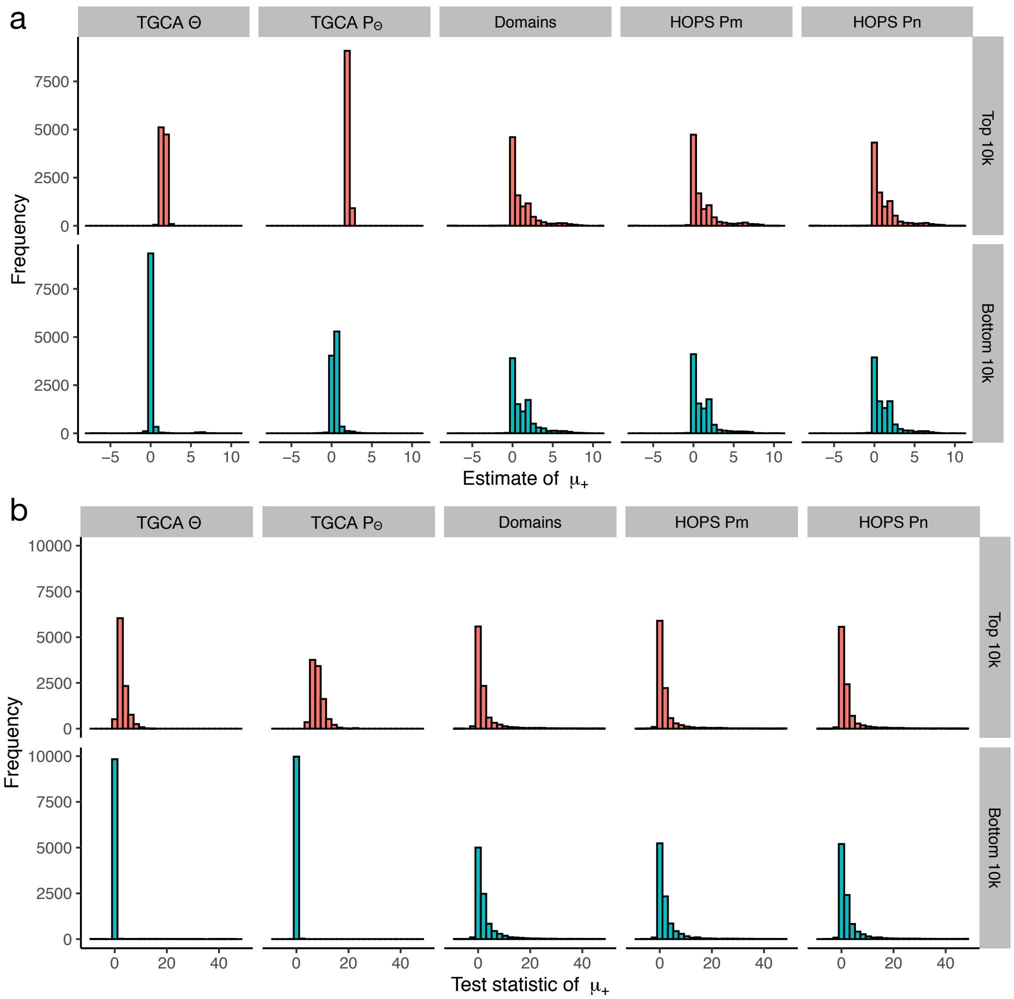


**Supplementary Figure 7: Comparison of $\hat{\mu}_+$ of top and bottom 10,000 SNPs ranked by five different statistics.** $P_\Theta$ values were derived from a two-sided Wald test against $\Theta = 0$. **a.** Distributions of $\mu_+$ estimates. **b.** Distributions of Wald test statistics against $\mu_+ = 0$. Source data are provided as a Source Data file.

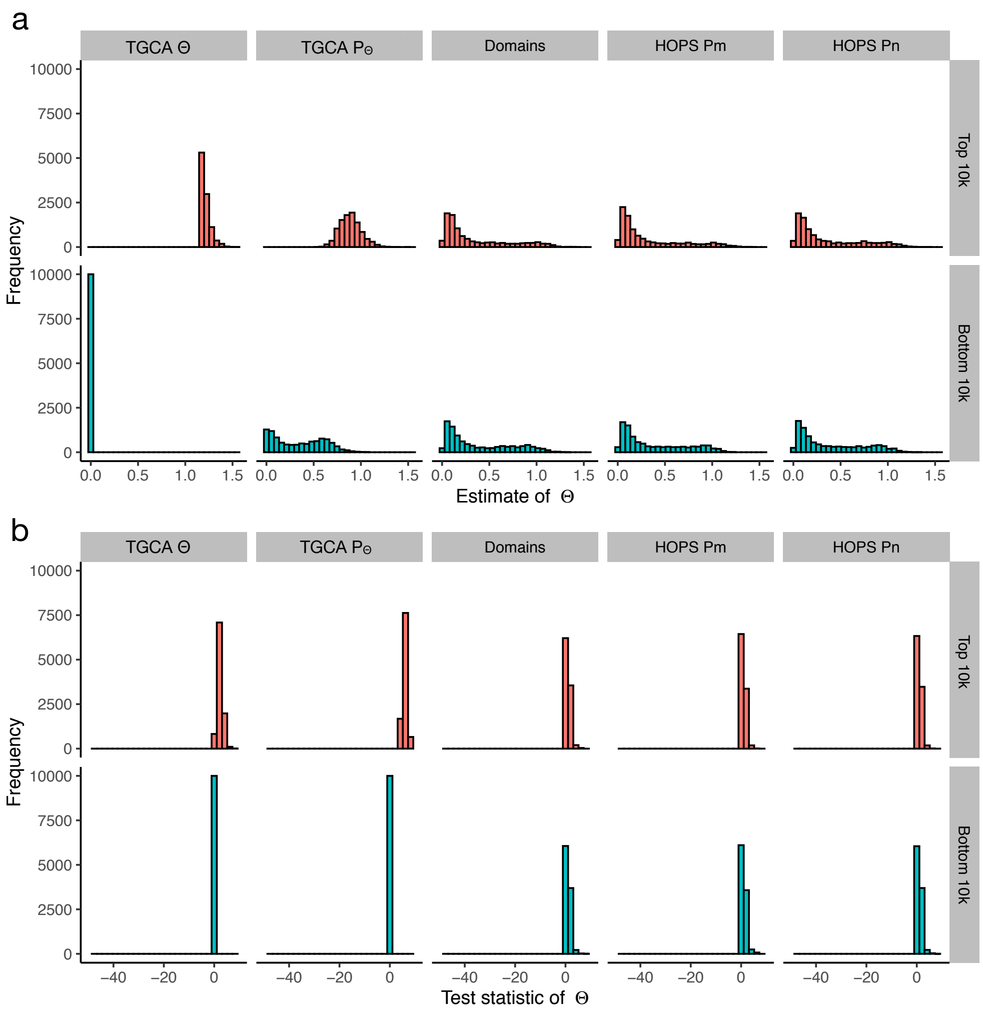


**Supplementary Figure 8: Comparison of $\hat{\Theta}$ of top and bottom 10,000 SNPs ranked by five different statistics.** $P_{\Theta}$ values were derived from a two-sided Wald test against $\Theta = 0$. **a.** Distributions of $\Theta$ estimates. **b.** Distributions of Wald test statistics against $\Theta = 0$. Source data are provided as a Source Data file.

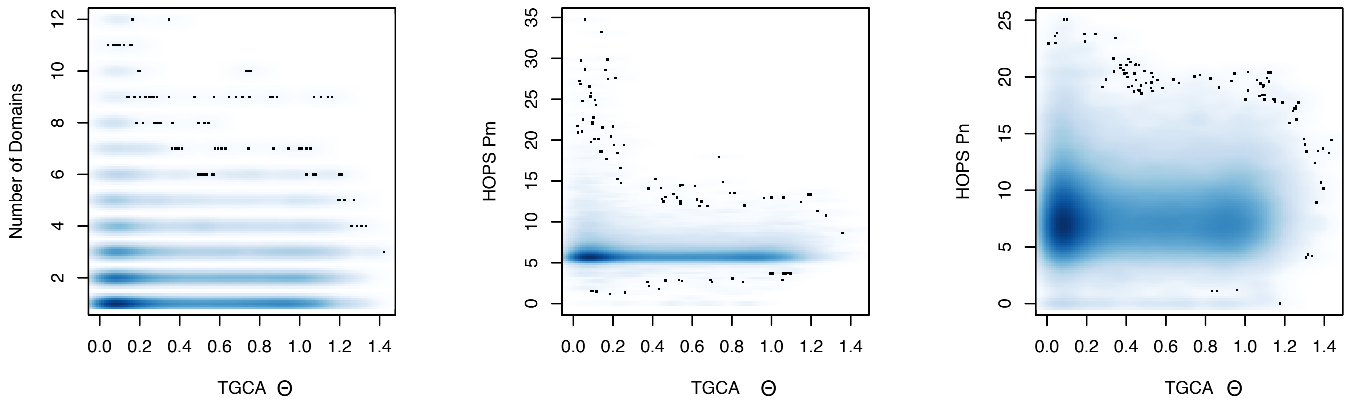


**Supplementary Figure 9: Comparison of TGCA with other quantities that measure pleiotropy.** Scatter plot shows the relationship between HOPS $P_m$, HOPS $P_n$, the number of associated phenotype domains and the estimate of TGCA $\Theta$ across 1,376 UK Biobank traits. Source data are provided as a Source Data file.

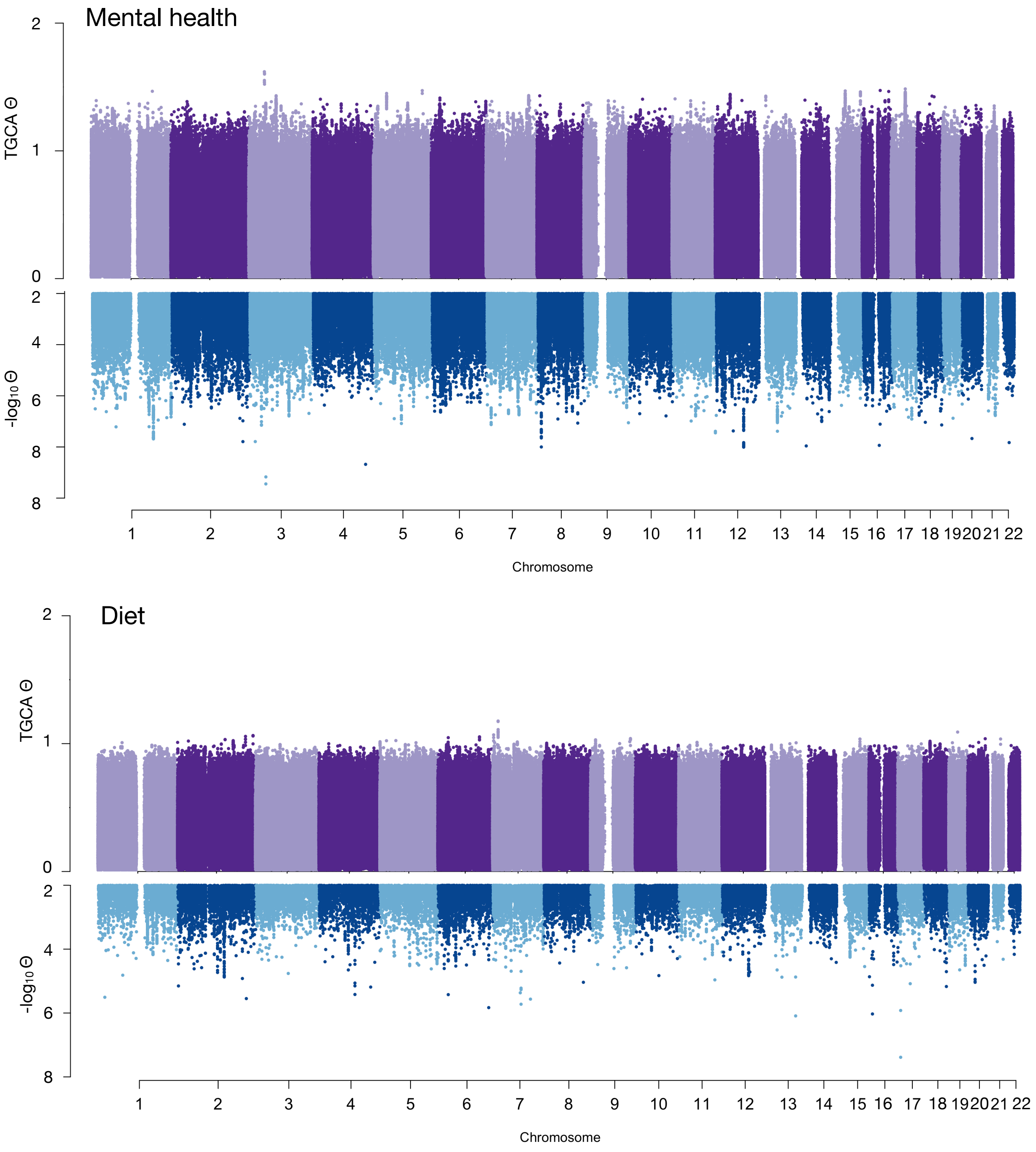


**Supplementary Figure 10: Genome-wide TGCA results in the other two trait domains.** The results correspond to 189 mental health traits and 139 diet-related traits in the UK Biobank. The estimated $\Theta$ are shown in purple and $-\log_{10} P$ for the null hypothesis $\Theta = 0$ in blue. Source

data are provided as genome-wide summary statistics in Data Availability.

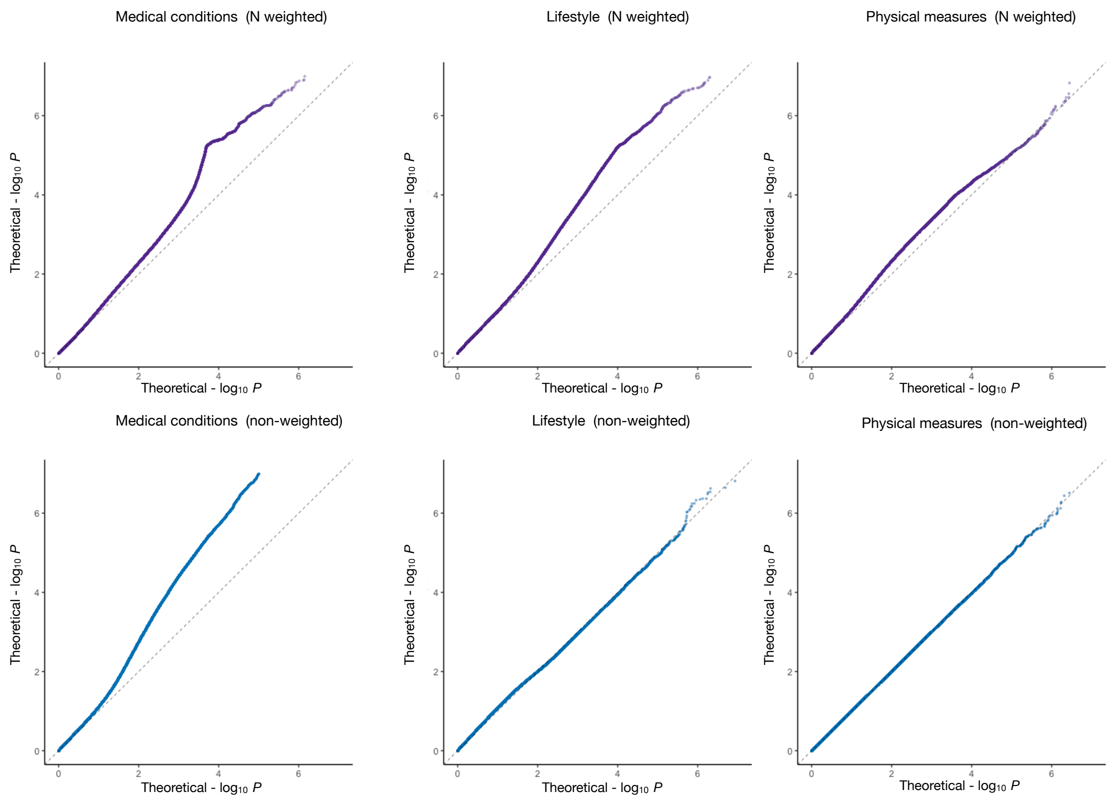


**Supplementary Figure 11: Quantile-quantile plot for genome-wide TGCA test statistics ($-\log_{10} P$) of medical conditions, lifestyle and physical measures traits.** Both the results re-weighted by sample sizes (purple) and without re-weighting (blue) are shown. Source data are provided as a Source Data file.

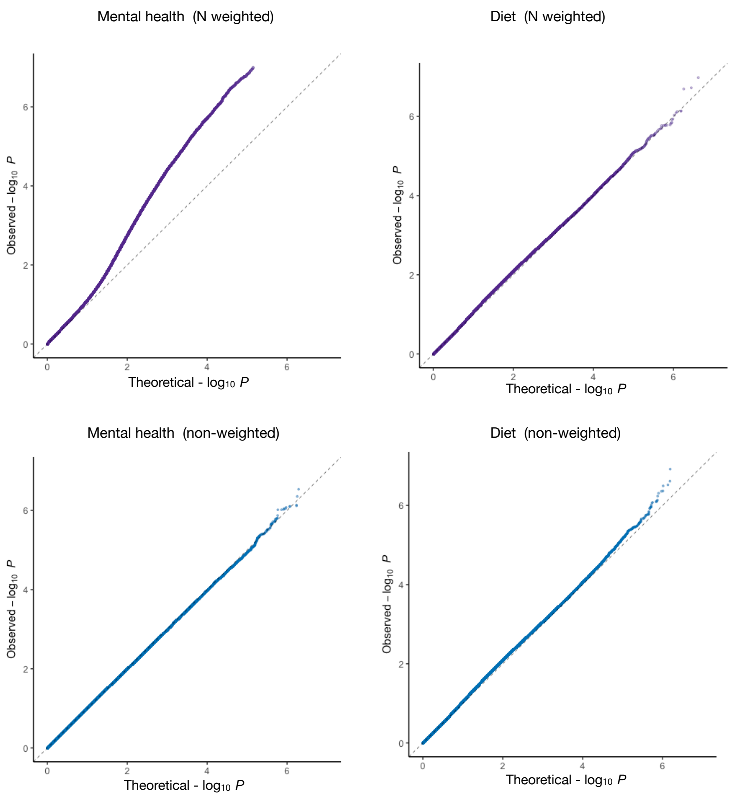


**Supplementary Figure 12: Quantile-quantile plot for genome-wide TGCA test statistics ($-\log_{10} P$) of mental health and diet traits.** Both the results re-weighted by sample sizes (purple) and without re-weighting (blue) are shown. Source data are provided as a Source Data file.

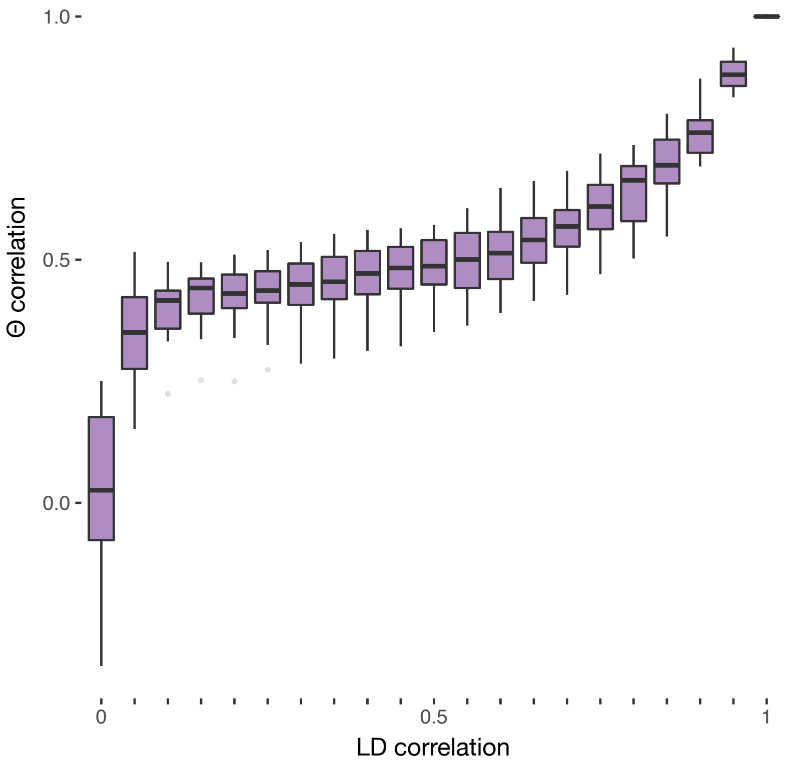


**Supplementary Figure 13: Relationship between the TGCA Θ estimate and the LD correlation between two SNPs.** For each LD correlation ($r = 0, 0.05, 0.1, \cdots, 1$), we repeated the simulation for 10 times, yielding 10 values to derive the statistics for each box plot. In each simulation, genotypes for two SNPs and 100,000 individuals were generated assuming the given LD correlation, and 200 independent true genetic effects corresponding to 200 phenotypes were drawn from $N(0, 1)$ and assigned to one of the two SNPs. We conducted an association analysis to obtain 200 Z-scores for the 200 traits for both SNPs and ran TGCA to estimate Θ. This procedure were repeated for 200 times to generate 200 pairs of $\hat{\Theta}$ to calculate the $\hat{\Theta}$ correlation between the two SNPs. Source data are provided as a Source Data file.

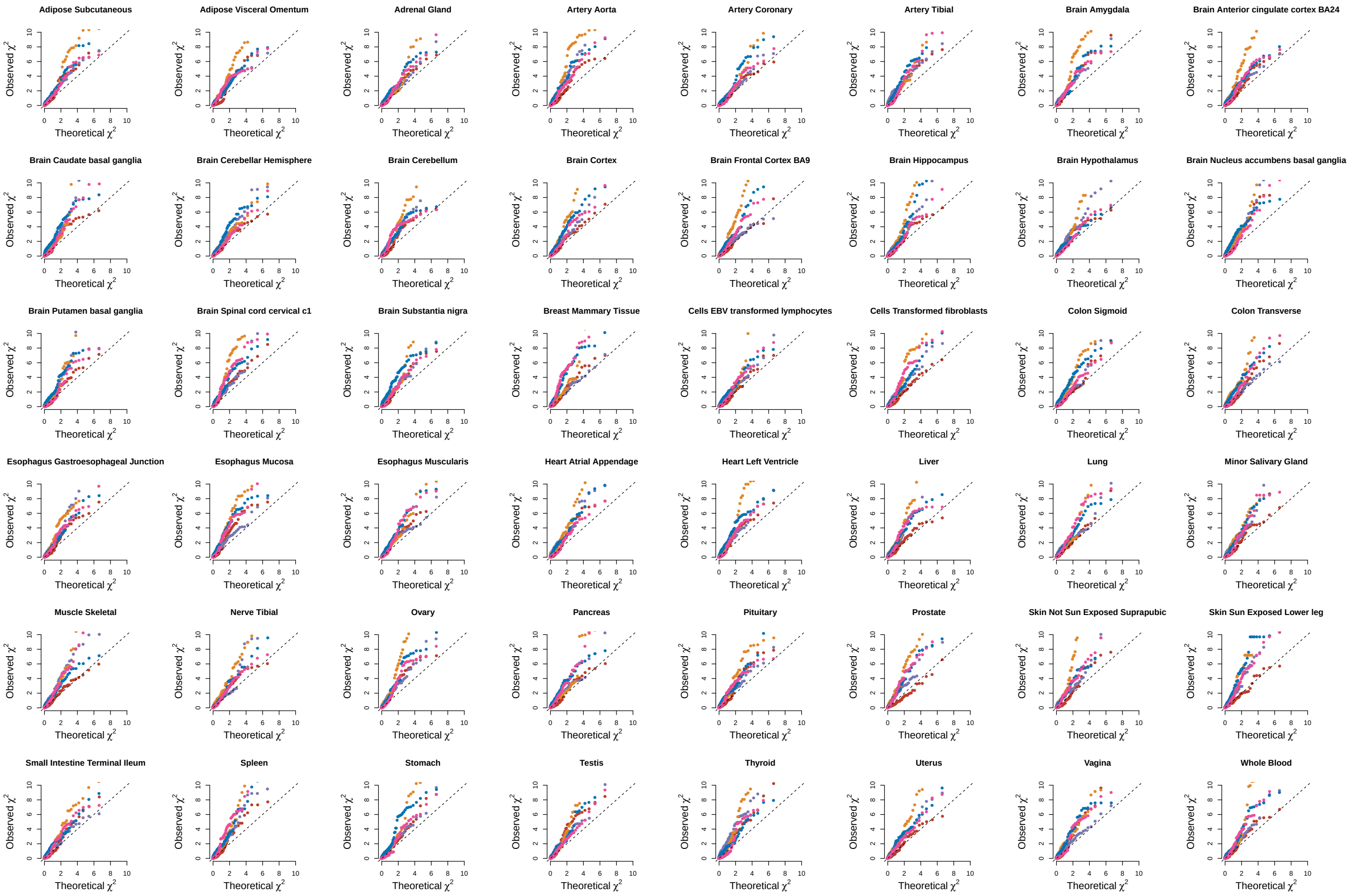


**Supplementary Figure 14: Distribution of TGCA statistic at eQTL of specifically expressed genes in 48 different tissues.** The quantile-quantile plots compare the observed TGCA $\chi^2$ statistics for $\hat{\Theta}$ for the five trait domains of physical measures (blue), mental health (yellow), medical conditions (pink), lifestyle (purple), and diet (red), at the cis-eQTL of the top 100 specifically expressed genes in each tissue. The expected null values were drawn from a $\chi^2(1)$ distribution. Source data are provided as a Source Data file.

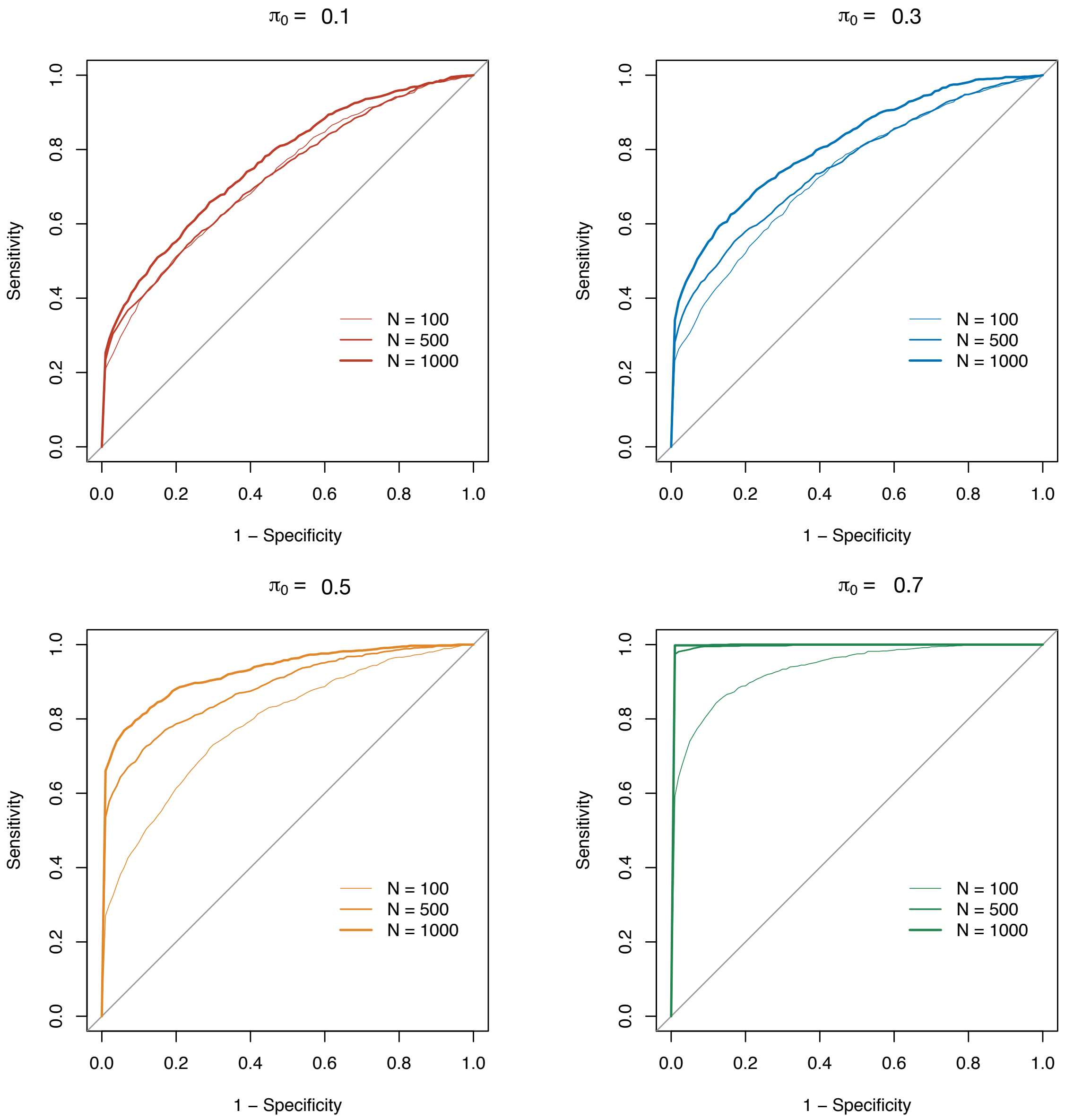


**Supplementary Figure 15: ROC curves of testing $\Theta = 0$ for different number of traits being modelled.** $K = 100$, 500, and 1000 independent Z-scores were simulated from a mixture distribution of $\pi_{-}N(\mu_{-}, \sigma_1^2) + \pi_0 N(0,1) + \pi_{+}N(\mu_{+}, \sigma_2^2)$. $\Theta$ had a true value of 1. The null effect proportion $\pi_0$ ranged from 0.1 to 0.7. Each curve was produced via 999 repeats of the simulation. Source data are provided as a Source Data file.

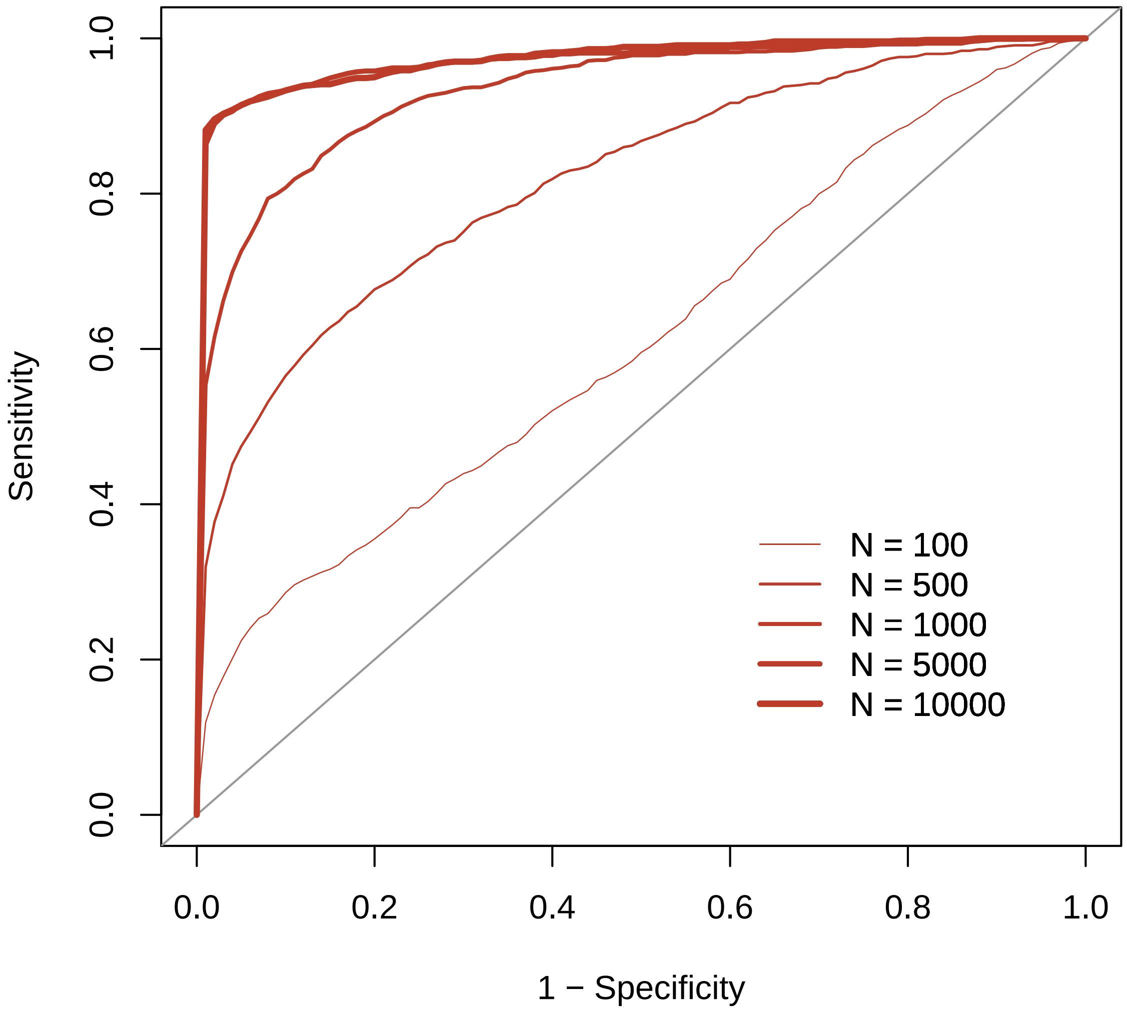


**Supplementary Figure 16: ROC curves of testing $\Theta = 0$ for different sample size of input GWAS summary statistics.** 200 independent true effects corresponding to 200 traits were simulated from a mixture Gaussian distribution $0.25N(-0.1, \sigma_1^2/100) + 0.5N(0, 0.01) + 0.25N(0.1, \sigma_2^2/100)$. The true values of the $\sigma^2$ parameters were randomly drawn from $\chi^2(1)$. Sample sizes of 100, 500, 1,000, 5,000, and 10,000 were used to simulate the genotypes and phenotypes based on a simple linear regression model. GWAS were then conducted to obtain 200 Z-scores for each sample size scenario, followed by TGCA estimation of $\Theta$. Each curve was produced via 999 repeats of the simulation. Source data are provided as a Source Data file.

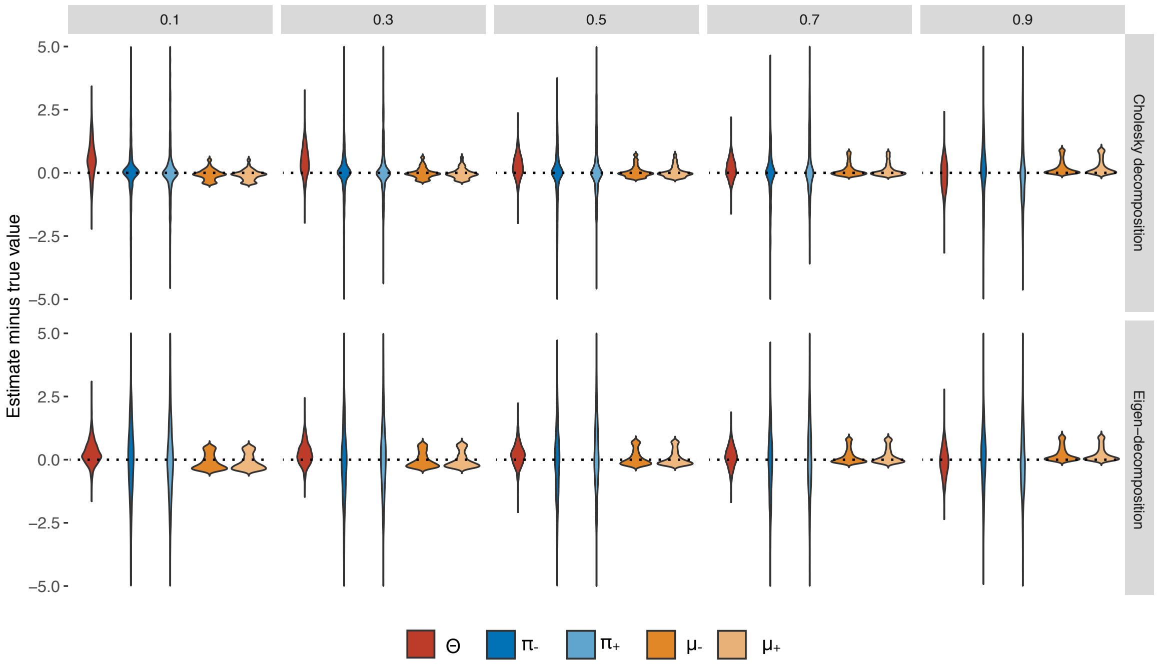


**Supplementary Figure 17: Comparison of simulation results for correlated traits using Cholesky and eigen-decomposition for decorrelation.** 122 correlated Z-scores for a single genetic variant were simulated from a mixture distribution with two non-null Gaussian components $\pi_- N(\mu_-, \sigma_1^2) + \pi_0 N(0,1) + \pi_+ N(\mu_+, \sigma_2^2)$, where $\pi_- = \pi_+$. The correlation structure were taken from the estimated phenotypic correlation matrix across 122 medical condition traits. Five different proportions of null effects $\pi_0$ were considered, ranging from 0.1 to 0.9. The 122 Z-scores were either decorrelated via Cholesky or eigen-decomposition. TGCA results using all the decorrelated data vectors were shown. The true values of $\mu_-$ and $\mu_+$ were drawn from -$|N(1,1)|$ and $|N(1,1)|$, respectively. $\sigma^2$'s were drawn from $\chi^2(1)$. The y-axis compares the estimated parameters with the true values. For each scenario, the simulation was repeated 999 times. Source data are provided as a Source Data file.

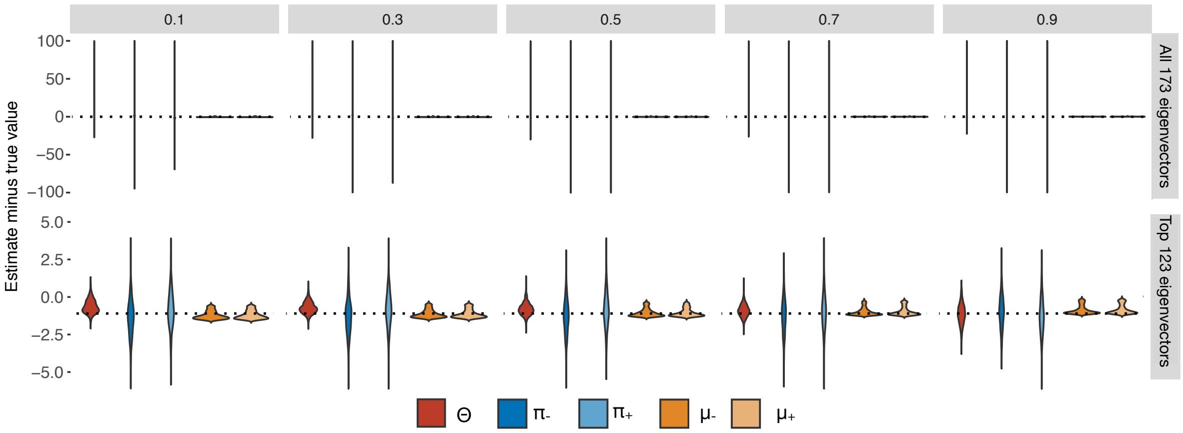


**Supplementary Figure 18: Comparison of simulation results for correlated traits with and without regularisation during the decorrelation procedure.** 173 correlated Z-scores for a single genetic variant were simulated from a mixture distribution with two non-null Gaussian components $\pi_- N(\mu_-, \sigma_1^2) + \pi_0 N(0,1) + \pi_+ N(\mu_+, \sigma_2^2)$, where $\pi_- = \pi_+$. The correlation structure were taken from the estimated phenotypic correlation matrix across 173 lifestyle traits. Five different proportions of null effects $\pi_0$ were considered, ranging from 0.1 to 0.9. The 173 Z-scores were decorrelated via eigen-decomposition. TGCA results using all the eigenvectors and the top 123 eigenvectors (explaining 90% information) were shown. The true values of $\mu_-$ and $\mu_+$ were drawn from -$|N(1,1)|$ and $|N(1,1)|$, respectively. $\sigma^2$'s were drawn from $\chi^2(1)$. The y-axis compares the estimated parameters with the true values. For each scenario, the simulation was repeated 999 times. Source data are provided as a Source Data file.

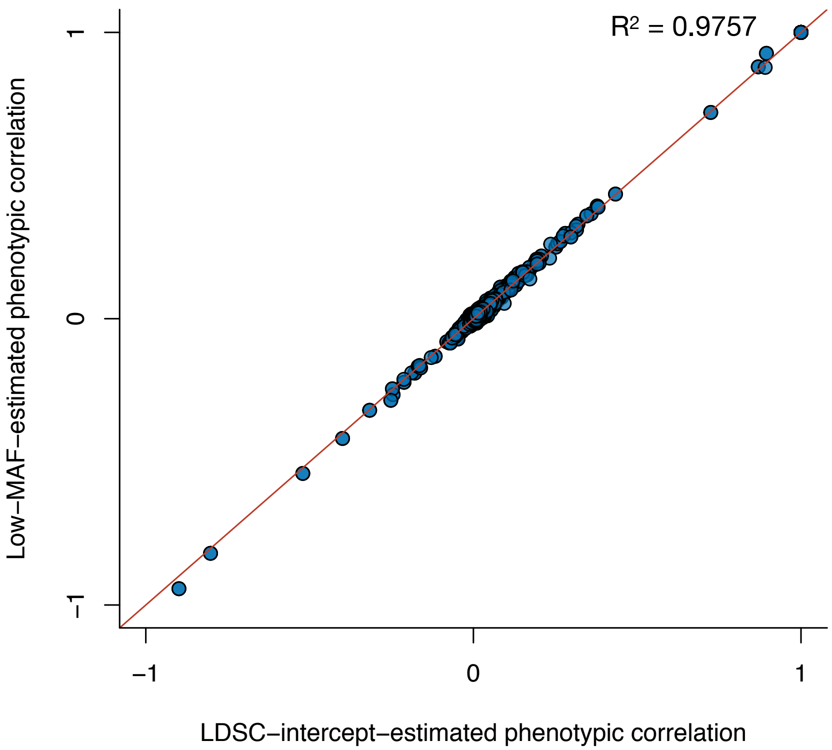


**Supplementary Figure 19: Comparison of phenotypic correlation estimation using low-MAF and LDSC-intercept estimators.** Phenotypic correlations across 122 medical conditions traits were estimated using the low-MAF method and LDSC-intercept, respectively. Source data are provided as a Source Data file.

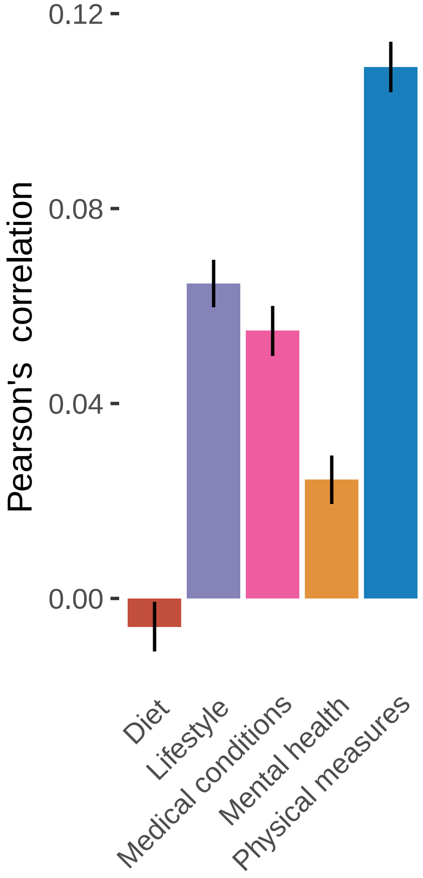


**Supplementary Figure 20: Correlations between estimated TGCA $\Theta$ in five traits domains and LD scores.** The LD scores were obtained from 1000 Genome Phase 3 European ancestry reference, pre-calculated in the ldsc software, where the MHC region was excluded. The error bars represent standard errors for the correlation estimates. The correlation estimates and standard errors were derived from 38,550 SNPs for diet traits, 42,085 SNPs for lifestyle traits, 40,333 SNPs for mental health traits, 36,917 SNPs for physical measures, and 37,917 SNPs for medical conditions. Source data are provided as a Source Data file.

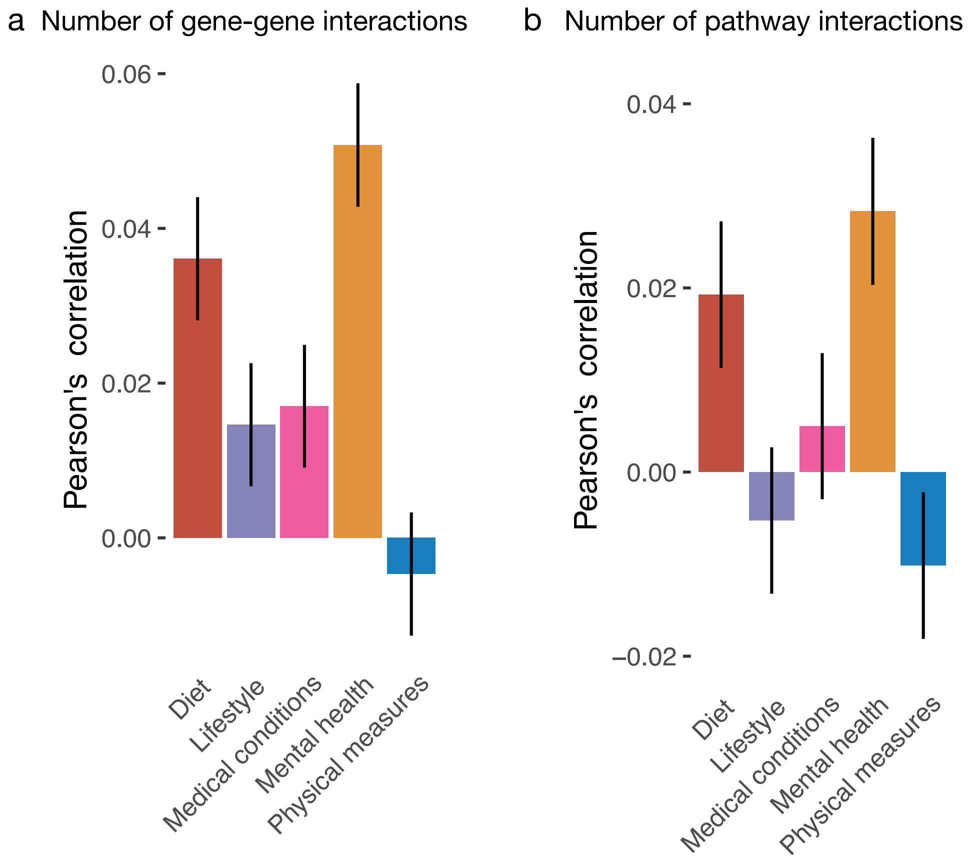


**Supplementary Figure 21: Correlations between gene-based $-\log_{10} P_{\Theta}$ for five traits domains and the number of gene-gene and pathway interactions.** The gene-based p-values for $\hat{\Theta}$ were computed through the MAGMA analysis procedure on FUMA. **a.** The number of genes that each gene interacts with was obtained from the GeneMANIA database. **b.** The number of pathways where each gene had at least one gene interacting with was obtained from the MSigDB database. The error bars represent standard errors for the correlation estimates. The correlation estimates and standard errors were derived from 15,767 genes for medical conditions, 15,816 genes for mental health traits, 15,650 genes for physical measures, 15,756 genes for diet traits, and 15,884 genes for lifestyle traits. Source data are provided as a Source Data file.

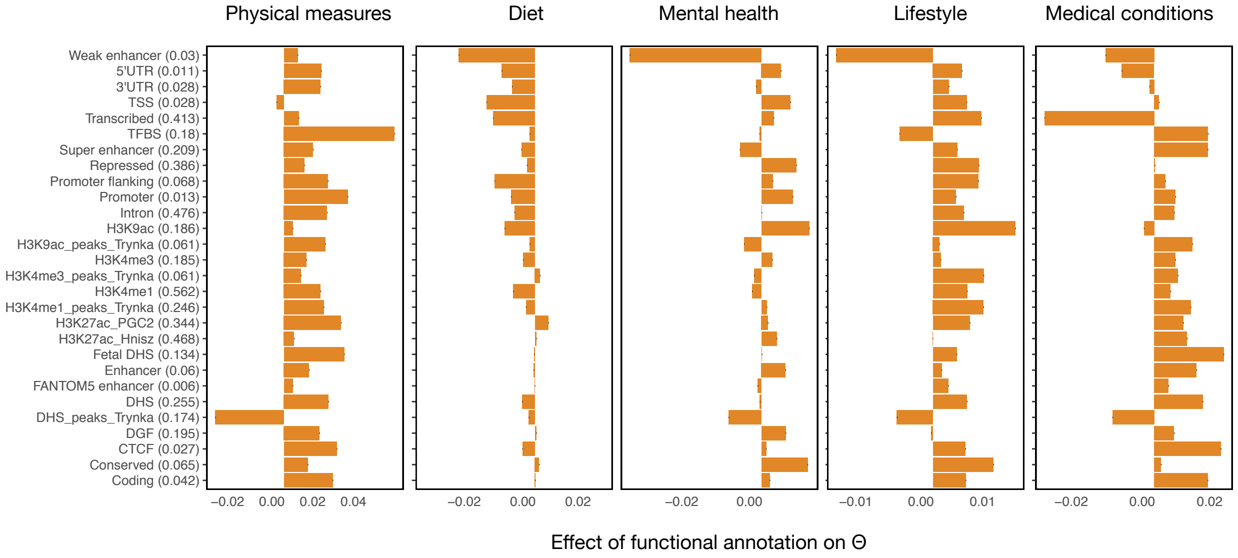


**Supplementary Figure 22: Association between TGCA estimated $\Theta$ for five trait domains and functional annotations.** The $\Theta$-annotation association was analysed using a linear regression of $\hat{\Theta}$ on the annotated SNPs, corrected for the LD scores of the SNPs. The median regression coefficients of the annotation variables across 100 sets of LD-pruned SNPs are plotted. The proportions of SNPs across the genome within the functional annotations are given in brackets. Source data are provided as a Source Data file.

# References

[1] Watanabe, K., Taskesen, E., van Bochoven, A. & Posthuma, D. Functional mapping and annotation of genetic associations with FUMA. *Nature Communications* **8**, 1826 (2017).

[2] de Leeuw, C. A., Mooij, J. M., Heskes, T. & Posthuma, D. MAGMA: generalized gene-set analysis of GWAS data. *PLoS computational biology* **11**, e1004219 (2015).

[3] Wang, K., Li, M. & Hakonarson, H. ANNOVAR: Functional annotation of genetic variants from high-throughput sequencing data. *Nucleic Acids Research* (2010).

[4] Liberzon, A. *et al.* The Molecular Signatures Database Hallmark Gene Set Collection. *Cell Systems* **1**, 417–425 (2015).

[5] Kutmon, M. *et al.* WikiPathways: Capturing the full diversity of pathway knowledge. *Nucleic Acids Research* (2016).

[6] Jordan, D. M., Verbanck, M. & Do, R. HOPS: a quantitative score reveals pervasive horizontal pleiotropy in human genetic variation is driven by extreme polygenicity of human traits and diseases. *Genome biology* **20**, 222 (2019).

[7] Finucane, H. K. *et al.* Partitioning heritability by functional annotation using genome-wide association summary statistics. *Nature Genetics* **47**, 1228–1235 (2015).

[8] Bulik-Sullivan, B. K. *et al.* LD Score regression distinguishes confounding from polygenicity in genome-wide association studies. *Nature Genetics* **47**, 291–295 (2015).

[9] Warde-Farley, D. *et al.* The GeneMANIA prediction server: biological network integration for gene prioritization and predicting gene function. *Nucleic Acids Research* **38**, W214–W220 (2010).

[10] Franz, M. *et al.* GeneMANIA update 2018. *Nucleic Acids Research* **46**, W60–W64 (2018).

[11] Liberzon, A. *et al.* Molecular signatures database (MSigDB) 3.0. *Bioinformatics* **27**, 1739–1740 (2011).
