# Supplementary material for: Total genetic contribution assessment across the human genome
Source: Nat Commun. 2021 May 14;12:2845. doi: 10.1038/s41467-021-23124-w (PMC8121943; doi:10.1038/s41467-021-23124-w)
Supplement: Supplementary file 3 — Description of Additional Supplementary Files [file 41467_2021_23124_MOESM3_ESM.pdf]

Description of Additional Supplementary Files

File Name: Supplementary Data 1.

Description: Lead loci for the UK Biobank trait domain of 122 medical conditions with TGCA $\Theta > 2$ The lead SNPs were identified by FUMA.

File Name: Supplementary Data 2.

Description: Lead loci for the UK Biobank trait domain of 117 physical measures with TGCA $\Theta > 2$. The lead SNPs were identified by FUMA.

File Name: Supplementary Data 3.

Description: Lead loci for the UK Biobank trait domain of 172 lifestyle phenotypes with TGCA $\Theta > 2$. The lead SNPs were identified by FUMA.

File Name: Supplementary Data 4.

Description: LDSC-based TGCA enrichment analysis in 48 tissues for five trait domains. Prop.SNPs: the proportion of SNPs in the top 10% of the specifically expressed genes of each tissue in the reference genome (1000 Genomes phase 3). Prop.h2: the estimated proportion of the heritability of the phenotype in each tissue annotation using stratified LD score regression. Enrichment = Prop.h2/Prop.SNPs. Enrichment.test.statistic represents whether the TGCA on specific annotations was higher than average (Enrichment = 1).

File Name: Supplementary Data 5.

Description: Regression-based TGCA enrichment analysis in 48 tissues for five trait domains. Estimate: the median of 100 regression coefficient estimates obtained through 100 subsets of LD-pruned SNPs. In each regression model, $\widehat{\Theta}$'s were regressed on each annotation indicator variable for tissue-specifically expressed genes, and the LD scores were used as a covariate.

File Name: Supplementary Data 6.

Description: Information of selected 1,511 UK Biobank phenotypes. phenotype: Unique phenotype identifier. description: Description of the phenotype. Variable type: type of phenotypic data: "categorical", "ordinal" or "continuous_irnt" (inverse-Gaussian transformed continuous phenotypes). source: Source of the phenotype. n_non_missing: Number of samples within the analysis, defined for the phenotype. n_missing: Number of samples within the analysis, missing for the phenotype. n_controls: For case/control phenotypes, number of controls. n_cases: For case/control phenotypes, number of cases. PHESANT_transformation: The transformations performed by PHESANT for the applicable phenotypes.

File Name: Supplementary Data 7.

Description: Phenotypic correlation of selected 1,376 QCed UK Biobank phenotypes.

File Name: Supplementary Data 8.

Description: Information of the 122 UK Biobank phenotypes categorised into medical conditions.

File Name: Supplementary Data 9.

Description: Information of the 189 UK Biobank phenotypes categorised into mental health traits.

File Name: Supplementary Data 10.

Description: Information of the 117 UK Biobank phenotypes categorised into physical measures.

File Name: Supplementary Data 11.

Description: Information of the 172 UK Biobank phenotypes categorised into lifestyle traits.

File Name: Supplementary Data 12.

Description: Information of the 139 UK Biobank phenotypes categorised into diet traits.

File Name: Supplementary Data 13.

Description: Gene-based TGCA of different traits domains and gene-gene/gene-pathway interaction results of corresponding genes. The gene-based p-values of the medical conditions domain were computed through the MAGMA analysis procedure on FUMA. SNPs were mapped to protein-coding genes if they were located within the annotated gene with 1 Kb window on each side. nGeneInteract: numbers of gene-gene interactions for each target gene in the GeneMANIA database. nPathwayInteract: numbers of pathways in the molecular signatures database (MSigDB) with at least one gene-gene interaction for each target gene.

File Name: Supplementary Data 14.

Description: Gene-based TGCA of different traits domains and gene-gene/gene-pathway interaction results of corresponding genes. The gene-based p-values of the mental health domain were computed through the MAGMA analysis procedure on FUMA. SNPs were mapped to protein-coding genes if they were located within the annotated gene with 1 Kb window on each side. nGeneInteract: numbers of gene-gene interactions for each target gene in the GeneMANIA database. nPathwayInteract: numbers of pathways in the molecular signatures database (MSigDB) with at least one gene-gene interaction for each target gene.

File Name: Supplementary Data 15.

Description: Gene-based TGCA of different traits domains and gene-gene/gene-pathway interaction results of corresponding genes. The gene-based p-values of the physical measures domain were computed through the MAGMA analysis procedure on FUMA. SNPs were mapped to protein-coding genes if they were located within the annotated gene with 1 Kb window on each side. nGeneInteract: numbers of gene-gene interactions for each target gene in the GeneMANIA database. nPathwayInteract: numbers of pathways in the molecular signatures database (MSigDB) with at least one gene-gene interaction for each target gene.

File Name: Supplementary Data 16.

Description: Gene-based TGCA of different traits domains and gene-gene/gene-pathway interaction results of corresponding genes. The gene-based p-values of the lifestyle trait domain were computed through the MAGMA analysis procedure on FUMA. SNPs were mapped to protein-coding genes if they were located within the annotated gene with 1 Kb window on each side. nGeneInteract: numbers of gene-gene interactions for each target gene in the GeneMANIA database. nPathwayInteract: numbers of pathways in the molecular signatures database (MSigDB) with at least one gene-gene interaction for each target gene.

File Name: Supplementary Data 17.

Description: Gene-based TGCA of different traits domains and gene-gene/gene-pathway interaction results of corresponding genes. The gene-based p-values of the diet trait domain were computed through the MAGMA analysis procedure on FUMA. SNPs were mapped to protein-coding genes if they were located within the annotated gene with 1 Kb window on each side. nGeneInteract: numbers of gene-gene interactions for each target gene in the GeneMANIA database. nPathwayInteract: numbers of pathways in the molecular signatures database (MSigDB) with at least one gene-gene interaction for each target gene.

File Name: Supplementary Data 18.

Description: Gene sets enrichment results from FUMA/GENE2FUNC procedure of the medical conditions domain. Hypergeometric tests are performed to test if mapped genes of the locus of the medical conditions domain are overrepresented in any of the pre-defined gene sets. Multiple testing correction is performed per category, i.e. canonical pathways, GO biological processes etc., separately. Gene sets were obtained from MsigDB, WikiPathways and reported genes from the GWAS catalog.

File Name: Supplementary Data 19.

Description: Gene sets enrichment results from FUMA/GENE2FUNC procedure of the physical measures domain. Hypergeometric tests are performed to test if mapped genes of the locus of the physical measures domain are overrepresented in any of the pre-defined gene sets. Multiple testing correction is performed per category, i.e. canonical pathways, GO biological processes etc.,

separately. Gene sets were obtained from MsigDB, WikiPathways and reported genes from the GWAS catalog.

File Name: Supplementary Data 20.

Description: Gene sets enrichment results from FUMA/GENE2FUNC procedure of the lifestyle trait domain. Hypergeometric tests are performed to test if mapped genes of the locus of the lifestyle trait domain are overrepresented in any of the pre-defined gene sets. Multiple testing correction is performed per category, i.e. canonical pathways, GO biological processes etc., separately. Gene sets were obtained from MsigDB, WikiPathways and reported genes from the GWAS catalog.
