# Supplementary material for: Total genetic contribution assessment across the human genome
Source: Nat Commun. 2021 May 14;12:2845. doi: 10.1038/s41467-021-23124-w (PMC8121943; doi:10.1038/s41467-021-23124-w)
Supplement: Supplementary file 5 — Reporting Summary [file 41467_2021_23124_MOESM5_ESM.pdf]

nature research

Corresponding author(s): Xia Shen

Last updated by author(s): Mar 15, 2021

Reporting Summary

Nature Research wishes to improve the reproducibility of the work that we publish. This form provides structure for consistency and transparency in reporting. For further information on Nature Research policies, see our Editorial Policies and the Editorial Policy Checklist.

Statistics

For all statistical analyses, confirm that the following items are present in the figure legend, table legend, main text, or Methods section.

n/a	Confirmed	
☐	☒	The exact sample size (*n*) for each experimental group/condition, given as a discrete number and unit of measurement
☒	☐	A statement on whether measurements were taken from distinct samples or whether the same sample was measured repeatedly
☐	☒	The statistical test(s) used AND whether they are one- or two-sided<br>*Only common tests should be described solely by name; describe more complex techniques in the Methods section.*
☐	☒	A description of all covariates tested
☐	☒	A description of any assumptions or corrections, such as tests of normality and adjustment for multiple comparisons
☐	☒	A full description of the statistical parameters including central tendency (e.g. means) or other basic estimates (e.g. regression coefficient) AND variation (e.g. standard deviation) or associated estimates of uncertainty (e.g. confidence intervals)
☐	☒	For null hypothesis testing, the test statistic (e.g. *F*, *t*, *r*) with confidence intervals, effect sizes, degrees of freedom and *P* value noted<br>*Give P values as exact values whenever suitable.*
☒	☐	For Bayesian analysis, information on the choice of priors and Markov chain Monte Carlo settings
☒	☐	For hierarchical and complex designs, identification of the appropriate level for tests and full reporting of outcomes
☐	☒	Estimates of effect sizes (e.g. Cohen's *d*, Pearson's *r*), indicating how they were calculated

Our web collection on statistics for biologists contains articles on many of the points above.

Software and code

Policy information about availability of computer code

Data collection	No software was used for data collection.
Data analysis	The main analysis in this study were carried out using R v3.5.1 from https://www.r-project.org/, with main software packages FUMA v1.3.5 at https://fuma.ctglab.nl/ and LDSC v1.0.1 from https://github.com/bulik/ldsc/. The code for the developed analysis method is available from https://github.com/xiashen/TGCA.

For manuscripts utilizing custom algorithms or software that are central to the research but not yet described in published literature, software must be made available to editors and reviewers. We strongly encourage code deposition in a community repository (e.g. GitHub). See the Nature Research guidelines for submitting code & software for further information.

Data

Policy information about availability of data

All manuscripts must include a data availability statement. This statement should provide the following information, where applicable:

- Accession codes, unique identifiers, or web links for publicly available datasets
- A list of figures that have associated raw data
- A description of any restrictions on data availability

The whole-genome summary statistics of TGCA for the five phenotype domains are available at the figshare repository https://doi.org/10.6084/m9.figshare.14216324. UK Biobank GWAS summary statistics are available at http://www.nealelab.is/uk-biobank/ukbround2announcement. Gene expression and eQTL data for 48 tissue types are available at: https://www.gtexportal.org/home/datasets. Source data are provided with this paper.

Field-specific reporting

Please select the one below that is the best fit for your research. If you are not sure, read the appropriate sections before making your selection.

- [x] Life sciences
- [ ] Behavioural & social sciences
- [ ] Ecological, evolutionary & environmental sciences

For a reference copy of the document with all sections, see nature.com/documents/nr-reporting-summary-flat.pdf

Life sciences study design

All studies must disclose on these points even when the disclosure is negative.

Sample size	No sample size was calculated, as the GWAS analysis were done prior to the statistical modeling.
Data exclusions	GWAS summary statistics for the genetic variants that did not pass the quality control procedure were excluded.
Replication	The study develops and evaluates an analysis method thus does not require a replication design for scientific discoveries.
Randomization	The study develops and evaluates an analysis method thus does not require a randomisation design for scientific discoveries. Furthermore, the data analysis is based on genome-wide association summary statistics, which measure genotype-phenotype associations, and the genotypes are already naturally randomized.
Blinding	The study develops and evaluates an analysis method thus does not require a blind design for scientific discoveries.

Reporting for specific materials, systems and methods

We require information from authors about some types of materials, experimental systems and methods used in many studies. Here, indicate whether each material, system or method listed is relevant to your study. If you are not sure if a list item applies to your research, read the appropriate section before selecting a response.

Materials & experimental systems

n/a	Involved in the study
x	Antibodies
x	Eukaryotic cell lines
x	Palaeontology and archaeology
x	Animals and other organisms
x	Human research participants
x	Clinical data
x	Dual use research of concern

Methods

n/a	Involved in the study
x	ChIP-seq
x	Flow cytometry
x	MRI-based neuroimaging
